# Allelic variations of intimin virulence gene (*eae*) in typical and atypical enteropathogenic *Escherichia coli* isolated from children with and without diarrhea in a rural area of southern Mozambique

**DOI:** 10.1186/s12879-026-13146-4

**Published:** 2026-03-25

**Authors:** Delfino Vubil, Arlindo Chidimatembue, Marcelino Garrine, Tacilta Nhampossa, Quique Bassat, Sozinho Acácio, Augusto Messa Jr., Inácio Mandomando

**Affiliations:** 1 Centro de Investigação em Saúde de Manhiça (CISM), PO BOX: 1929, Rua 12, Cambeve, Vila da Manhiça, Maputo, Mozambique; 2 Instituto Nacional de Saúde (INS), Marracuene District, Maputo, Mozambique; 3 ISGlobal, Barcelona, Spain; 4 Facultat de Medicina i Ciències de la Salut, Universitat de Barcelona (UB), Barcelona, Spain; 5 ICREA, Pg. Lluís Companys 23, Barcelona, 08010 Spain; 6 Institut Clínic de Medicina I Dermatologia, Hospital Clínic de Barcelona, Barcelona, Spain; 7 Pediatrics Department, Hospital Sant Joan de Déu, Universitat de Barcelona, Esplugues, Barcelona, Spain; 8 CIBER de Epidemiología y Salud Pública, Instituto de Salud Carlos III, Madrid, Spain; 9 Global Health and Tropical Medicine (GHTM), Associate Laboratory in Translation and Innovation Towards Global Health (LA-REAL), Instituto de Higiene e Medicina Tropical (IHMT), Universidade NOVA de Lisboa, UNL, Lisbon, Portugal

**Keywords:** EPEC, Virulence genes, Alleles, Diarrhea, Mozambique

## Abstract

**Background:**

Enteropathogenic *Escherichia coli* (EPEC) is among the major causes of childhood diarrhea in low- and middle-income countries. The pathogenicity of EPEC strains has been associated with the expression of diverse virulence genes, therefore, it is possible that different EPEC isolates possess different pathogenic potentialities. However, there are limited data about the allelic distribution of EPEC critical virulence genes, especially in African settings.

**Methods:**

Herein, we assessed the molecular variability of the intimin virulence gene (*eae*) in EPEC isolates, recovered from children under 5 years in a case-control study of diarrheal disease in Manhiça, southern Mozambique, from 2007 to 2010. A total number of 157 EPEC isolates (53 cases of diarrhea and 104 controls) were characterized. The identification of EPEC and typing of its virulence genes was accomplished by conventional PCR. We compared the different intimin alleles within cases and controls, and clinically characterized the cases.

**Results:**

Typical EPEC (*eae* with *bfpA*) was the most predominant with 71.9% (113/157), while atypical EPEC (*eae* only) was detected in 26.1% (41/157). Eighteen different intimin types were identified, with *eae*-λ (28.0%) the most frequently detected, followed by *eae*-β1 (24.8%) and *eae*-ξR/β2B‡§ (19.7%), although there were no statistically significant differences between cases and controls, suggesting a possible role of other virulence factors. When analyzing the potential implication of the intimin alleles with clinical characteristics, we found that *eae*-λ and *eae*-β1 were mostly detected in children with fever and vomiting, additional investigations are warranted.

**Conclusions:**

Our findings indicate a high diversity of intimin alleles among EPEC diarrheal isolates from a rural area in southern Mozambique. This is the first report on the molecular diversity of the EPEC intimin gene in Mozambique.

## Introduction

Despite extensive efforts to reduce childhood deaths due to diarrheal disease, it remains the second leading cause of infant mortality in low- and middle-income countries (LMIC) [1, 2]. A wide range of enteric pathogens, such as viruses, bacteria, and parasites, are recognized as the causes of the disease. In such settings, Enteropathogenic *Escherichia coli* (EPEC) is among the major causes of childhood diarrhea [3, 4].

EPEC strains are mainly characterized by their capacity to closely adhere to intestinal epithelial cells and induce attaching and effacing (AE) lesions [5]. The AE phenotype is defined by genes from the locus for enterocyte effacement (LEE), a 35 kb pathogenicity island found in the bacterial genome [6]. This island consists of around 41 open reading frames [7] and encodes elements of a type III secretion system, several effector proteins, and the intimin adhesion [7, 8]. Intimin is essential in the formation of AE lesions and is codified by the highly polymorphic *eae* gene, with more than 25 allelic variants [9]. The identification of intimin alleles serves as a valuable method for typing of EPEC and STEC in diagnostics as well as in studies of pathogenesis, epidemiology, clonal analysis, and immunology. Furthermore, it has been proposed that intimin variants are associated with different cell tropism in host tissues [6], however, functional studies showed that the determination of host and tissue specificity by AE-lesion-forming *E. coli* is multifactorial, involving both intimin and other bacterial and host determinants [10]. While numerous intimin variants exist, the most prevalent types include alpha (α), beta (β), and gamma (γ), however, the distribution can vary according to *E.coli* strain, host, and geographical location [11]. Intimin α has been associated with EPEC, intimin γ with Enterohemorrhagic *E.coli* (EHEC), while intimin β appears to be more ubiquitous and is found across human and animal pathogens, including EPEC [10, 12].

On the other hand, EPEC strains are divided into typical and atypical, according to their ability to form microcolonies on the intestinal epithelial cell surface. This phenotype, known as localized adherence (LA), is associated with a significant virulence plasmid identified as EPEC adherence factor (EAF) [5]. This plasmid contains two key operons (*bfp and per*). The *bfp* operon codifies the type IV bundle-forming pilus (Bfp), while *per* encodes the transcriptional activator referred to as plasmid-encoded regulator (Per), which is involved in the complete virulence of EPEC [13]. While Bfp facilitates the attachment of EPEC to the epithelial cells and creation of microcolonies, Per is necessary for the optimal activation and functioning of LEE-encoded genes and the expression of Bfp [14, 15]. The identification of EPEC and its classification into typical and atypical strains relies on the detection of *eae* and *bfpA* genes [5]. *E. coli* strains exhibiting the AE genotype (*eae*) and that contain the EAF plasmid (*bfpA*) are classified as typical EPEC, whereas strains with the AE genotype lacking the EAF plasmid (*bfpA*) are categorized as atypical EPEC [5].

Typical EPEC has been acknowledged for many years as the main cause of gastroenteritis in infants in LMIC, while atypical EPEC was more frequent in developed countries [16]. Currently, several studies have reported high frequencies of atypical EPEC in LMIC [17–19]. The dominance of atypical EPEC was further observed in the VIDA study (Vaccine Impact on Diarrheal in Africa) conducted from 2015 to 2018 in Mali, The Gambia, and Kenya [4]. These changes in the epidemiology of EPEC reinforce the need for continuous surveillance to track disease evolution. Furthermore, there are limited studies investigating the allelic distribution of EPEC in African settings or if a particular allele is associated with a specific clinical feature. In this study we aimed to identify the allelic variants of the *eae* gene and potential association with clinical characteristics in a collection of EPEC isolates, recovered from children under 5 years old in the Manhiça District (Southern Mozambique), as part of the Global Enteric Multicenter Study (GEMS) [20]. This case-control study evaluated the burden, microbiological etiology and clinical presentation of childhood moderate-to-severe diarrhea (MSD) in sub-Saharan Africa and Southeast Asia. While, EPEC was not associated with MSD in the GEMS study, the children infected with EPEC had a high risk of death [20]. Therefore, the data from this study could shed light on the most prevalent *eae* alleles and the possible relation with clinical characteristics or outcomes.

## Materials and methods

### Bacterial isolates and study population

All isolates analyzed were collected within the Global Enteric Multicenter Study (GEMS) [20], a case-control study of MSD conducted from December 2007 to December 2010 in sub-Saharan Africa and Southeast Asian countries. In Mozambique, the study was conducted by the *Centro de Investigação em Saúde de Manhiça* (CISM), in the Manhiça district, a rural area of Maputo province in southern Mozambique. All methods for study design and participant enrollment have been described elsewhere [21]. Briefly, in the GEMS, the cases of diarrhea were all children under 5 years presenting to the selected health care facilities with MSD upon seeking consent from the mother or caretaker. While controls were community healthy children, matched by age, sex and neighborhood of the index case, using the Health and Demographic Surveillance System (HDSS) [22]. Demographic and clinical information, such as age, sex, diarrheal symptoms, breastfeeding patterns, water and sanitation, were recorded throughout the study period. Stool samples were collected for both cases of diarrhea and controls [20]. For bacterial isolation, samples were cultured in different microbiological culture media. *E. coli* was identified by colony morphology and biochemical tests upon culturing in MacConkey and Xylose Lysine Deoxycholate (XLD) [23]. Three single colonies of *E. coli* were stored in Triple Soy broth (TSB) + glycerol at -80 °C for the detection of diarrheagenic *E.coli* and further analyses.

### Molecular detection of EPEC, ETEC and EAEC

Molecular detection of EPEC, including Enteroaggregative *E.coli* (EAEC) and Enterotoxigenic *E.coli* (ETEC) was performed by multiplex PCR as described elsewhere [23]. The target genes for EPEC included the intimin (*eae* gene) outer membrane protein adhesion and the EPEC plasmid-encoded bundle-forming pilus (BFP); for ETEC, the heat-labile enterotoxin (*eltB*) and heat-stable enterotoxin (*estA*), while the detection of EAEC was based on the presence of the plasmid-encoded gene *aatA* and the EAEC chromosomally encoded *aaiC* locus. Table 1 shows the detailed information regarding the primers used and the amplicon product size. Strains with both *eae* and *bfpA* genes were classified as typical EPEC, while those with *eae* only were deemed as atypical EPEC [5].

**Table 1:** Primers sequence and amplicon product size for detection of diarrheagenic *E.coli*

Pathogen	Primer	Target gene	Primer sequence (5’-3’)	Amplicon (bp)
ETEC	LT-F	*elt*	CACACGGAGCTCCTCAGTC	508
	LT-R		CCCCCAGCCTAGCTTAGTTT	
	ST-F	*est*	GCTAAACCAGTAG/AGGTCTTCAAAA	147
	ST-R		CCCGGTACAG/AGCAGGATTACAACA	
EPEC	BFPA-F	*bfpA*	GGAAGTCAAATTCATGGGGG	367
	BFPA-R		GGAATCAGACGCAGACTGGT	
	EAE-F	*eae*	CCCGAATTCGGCACAAGCATAAGC	881
	EAE-R		CCCGGATCCGTCTCGCCAGTATTCG	
EAEC	CVD432F	*aatA*	CTGGCGAAAGACTGTATCAT	630
	CVD432R		CAATGTATAGAAATCCGCTGTT	
	AAIC F	*aaiC*	ATTGTCCTCAGGCATTTCAC	215
	AAIC R		ACGACACCCCTGATAAACAA	

For DNA extraction, the isolates were recovered from − 80 °C freezer, streaked on MacConkey agar plates, and incubated for 18–24 h in a 37 °C incubator. A loop full of bacterial colonies was suspended in 500 µL of distilled water and boiled for 10 min in a heat block, and then centrifuged at 2500 RCF for 10 min. Three µL of the supernatant was used as the DNA template for PCR. DNA amplification was performed in a 20 µL reaction volume, composed of 2.5 µL of 10X PCR buffer with 2 mM of MgCl_2_ (New England Biolabs, Ipswich, MA, USA), 2 µL of 10 mM deoxynucleotide triphosphate (DNTPs) (Fermentas, Waltham, Massachusetts, USA), 0.4 µL of 20 pmol/µl of each primer, 0.25 µL of Taq DNA polymerase (5U/µl, New England Biolabs, Ipswich, MA, USA), and 7. 7 µL of RNAse-free water. The PCR was amplified using the following conditions: pre-heating at 96 °C for 4 min, followed by 35 cycles of denaturation at 95 °C for 20 s, annealing at 57 °C for 20 s, elongation at 72 °C for 1 min, and final extension at 72 °C for 7 min. The amplified product was visualized in 2% agarose gel after staining with ethidium bromide.

### Molecular typing of *eae* alleles

All *eae* positive samples were subtyped for 19-allele variability with specific primers as described elsewhere [24]. DNA amplification was performed in a 20 µL reaction volume composed of 12 µL of PCR master mix 2X (Qiagen Multiplex PCR kit), 0.25 µM of each primer (0.5 µL), 4 µL of PCR water (nuclease-free), and 3 µL of DNA template. The PCR conditions were as follows: pre-heating at 95 °C for 5 min, followed by 35 cycles of denaturation at 94 °C, for 1 min, annealing at 55–66 °C for 1 min, elongation at 72 °C for 2 min, and final extension at 72 °C for 10 min. The annealing temperature was adjusted accordingly for each primer set [24] (Table 2). The amplified product was visualized in 2% agarose gel after staining with ethidium bromide.

**Table 2:** Primers sequence for typing of *eae* alleles

Gene	Primer	Oligonucleotide sequence 5’-3’	Fragment size (bp)	Annealing Temperature
*eae*-α1	EAE-FB	AAAACCGCGGAGATGACTTC	820	60
	EAE-A	CACTCTTCGCATCTTGAGCT		
*eae*-α2	IH2498aF	AGACCTTAGGTACATTAAGTAAGC	517	60
	IH2498aR	TCCTGAGAAGAGGGTAATC		
*eae*-β1	B1F	CACAATTAATGCACCGGGT	241	55
	B1R	GCTTGATACACCTGATGACT		
*eae*-ξR/β2B‡§	B2F	TGAAGGGGGGAACCCCTGTG	564	62
	B2R	TTTCTTTTGACTGTGCTAAAGC		
*eae*-δ/k/β2O||	EAE-FB	AAAACCGCGGAGATGACTTC	830	60
	EAE-D	CTTGATACACCCGATGGTAAC		
*eae*-γ1	EAE-FB	AAAACCGCGGAGATGACTTC	804	60
	EAE-C1	AGAACGCTGCTCACTAGATGTC		
*eae*-θ/γ2	EAE-C2F	AGAACGTTACTGGTGACTTA	414	58
	EAE-C2R	CTGATATTTTATCAGCTTCA		
*eae*-ε1	EAE-FB	AAAACCGCGGAGATGACTTC	722	66
	LP5	AGCTCACTCGTAGATGACGGCAAGCG		
*eae*-νR/ε2§	EAE-E2F	AATACAGAAGTTAAGGCAT	378	58
	EAE-E2R	ACGACCACTATTCATTTC		
*eae*-ζ	Z1	GGTAAGCCGTTATCTGCC	206	62
	Z2	ATAGCAAGTGGGGTGAAG		
*eae*-η1/η2	EAE-FB	AAAACCGCGGAGATGACTTC	702	60
	ETA-B	TAAGCGACCACTATTCGTG		
*eae*-η1	ETA-FN	CGCTTTGTTTAATGCCGATAAA	410	62
	ETA-RN	GACTGCGTAATGCACTG		
*eae*-ι1	EAE-FB	AAAACCGCGGAGATGACTTC	651	55
	IOTA-B	GTCATATTTAACTTTTACACTA		
*eae*-µR/ι 2§	Iota2-F	CTGGTAAAGCGATAGTCAAAC	936	58
	Iota2-R	GCGTTTTTGAAGAAACATTTTGC		
*eae*- λ	68.4 F	CGGTCAGCCTGTGAAGGGC	370	64
	68.4R	AATACCGGAAGAGGCATCTAT		
*eae*-µB#	EAE-FB	AAAACCGCGGAGATGACTTC	655	60
	FV373R	ACTCATCATAATAAGCTTTTTGG		
*eae*-νB#	IH1229aF	CACAGCTTACAATTGATAACA	311	60
	IH1229aR	CTCACTATAAGTCATACGACT		
*eae*-ξB#	EAE-FB	AAAACCGCGGAGATGACTTC	468	66
	B49R	ACCACCTTTAGCAGTCAATTTG		
*eae*-o	H2997fF	AGCGTTAGCAATGCCGCAGTTGAT	271	60
	IH2997fR	CAACGGTAATTGTTGTTTCC		

### Data analysis

The clinical and laboratory data were combined to form a unique dataset, and all the analyses were conducted with STATA 14.2 (StataCorp, College Station, TX, USA). We compared the different intimin alleles against cases, controls, and clinical characteristics of the children. The proportions of the different intimin alleles were compared using Fisher’s exact test with α = 0.05.

## Results

### Characteristics of children with EPEC

Between December 2007 and December 2010, 1978 children younger than 5 years with and without diarrhea were enrolled in Mozambique, as part of the GEMS study. EPEC was isolated in 9.3% (183/1978) of children, from which 157/183 (85.7%) viable isolates were characterized in this study (53 from diarrheal cases and 104 from the controls). In terms of age, most of the isolates were recovered from children aged 0–11 months (64.3%; 101/157), followed by 12–23 months (22.2%; 35/157) and 24–59 months (13.3%; 21/157). Typical EPEC was more common, with 71.9% (113/157), compared to atypical EPEC, with 26.1% (41/157) (Table 3). Three isolates (1.9%) were positive for the *bfpA* gene only, which does not fall into the typical nor atypical EPEC classification. The proportions of the other pathotypes were 202/1978 (10.2%) for ETEC and 535/1978 (27.0%) for EAEC.

**Table 3:** Proportion of EPEC isolates in cases of diarrhea and matched controls, according to age strata

All EPEC			
Age (months)	Cases of diarrhea *n* (%)	Control group *n* (%)	Total *n* (%)
0–11	35 (66.0)	66 (63.4)	101 (64.3)
12–23	10 (18.8)	25 (24.0)	35 (22.2)
24–59	8 (15.1)	13 (12.5)	21 (13.3)
**All ages**	**53 (33.7)**	**104 (66.2)**	157* (100)
**Typical EPEC**			
**Age (months)**	**Cases of diarrhea n (%)**	**Control group n (%)**	**Total n (%)**
0–11	26 (68.4)	52 (69.3)	78 (69.0)
12–23	7 (18.4)	16 (21.3)	23 (20.3)
24–59	5 (13.1)	7 (9.3)	12 (10.6)
** All ages**	**38 (71.6)**	**75 (72.1)**	**113 (71.9)**
**Atypical EPEC**			
**Age (months)**	**Cases of diarrhea n (%)**	**Control group n (%)**	**Total n (%)**
0–11	9 (60.0)	13 (50.0)	22 (53.6)
12–23	3 (20.0)	8 (30.7)	11 (26.8)
24–59	3 (20.0)	5 (19.2)	8 (19.5)
** All ages**	**15 (28.3)**	**26 (25)**	**41 (26.1)**

* Includes three samples positive to *bfpA* only, which do not fall into EPEC classification

### Typing of the intimin (*eae*) virulence gene

The majority of the *eae*-positive isolates were typable, 85% (131/154), with a total number of 18 different *eae*-alleles variants detected out of the 19 screened. *eae*-λ (28.0%) was the most frequent allele, followed by *eae*-β1 (24.8%) and *eae*-ξR/β2B‡§ (19.7%) (Table 4), however, there were no statistical differences between cases of diarrhea and controls. Typical EPEC had more *eae* allelic diversity (all 18 alleles detected) compared to atypical EPEC (only 8 alleles detected). Of the non-type isolates, 17 were from children in the control group and 6 from cases of diarrhea.

**Table 4:** Intimin typing in typical and atypical EPEC

Intimin types	Typical EPEC		Atypical EPEC		All EPEC		Total
	Diarrheal samples (38)	Control samples (75)	Diarrheal samples (15)	Control samples (26)	Diarrheal Samples (53)	Control samples (101)	154
	*n* (%)	*n* (%)	*n* (%)	*n* (%)	*n* (%)	*n* (%)	*n* (%)
*eae*-α1	1 (2.6)	5 (6.6)	0 (0.0)	1 (3.8)	1 (1.8)	6 (5.9)	7 (4.5)
*eae*-α2	1 (2.6)	2 (2.6)	0 (0.0)	1 (3.8)	1 (1.8)	3 (2.9)	4 (2.5)
*eae*-β1	**6 (15.7)**	**17 (22.6)**	**7 (46.6)**	**9 (34.6)**	**13 (24.5)**	**26 (25.7)**	**39 (25.3)**
*eae*-ξR/β2B‡§	**7 (18.4)**	**17 (22.6)**	**4 (26.6)**	**3 (11.5)**	**11 (20.7)**	**20 (19.8)**	**31 (20.1)**
*eae*-δ/k/β2O||	1 (2.6)	2 (2.6)	0 (0.0)	0 (0.0)	1 (1.8)	2 (1.9)	3 (1.9)
*eae*-γ1	0 (0.0)	2 (2.6)	0 (0.0)	0 (0.0)	0 (0.0)	2 (1.9)	2 (1.2)
*eae*-θ/γ2	2 (5.2)	2 (2.6)	2 (13.3)	2 (7.6)	4 (7.5)	4 (3.9)	8 (5.1)
*eae*-ε1	2 (5.2)	4 (5.3)	0 (0.0)	1 (3.8)	2 (3.7)	5 (4.9)	7 (4.5)
*eae*-νR/ε2§	1 (2.6)	1 (1.3)	0 (0.0)	0 (0.0)	1 (1.8)	1 (0.9)	2 (1.2)
*eae*-ζ	1 (2.6)	6 (8.0)	0 (0.0)	1 (3.8)	1 (1.8)	7 (6.9)	8 (5.1)
*eae*-η1/η2	1 (2.6)	4 (5.3)	0 (0.0)	0 (0.0)	1 (1.8)	4 (3.9)	5 (3.2)
*eae*-η1	1 (2.6)	8 (10.6)	0 (0.0)	0 (0.0)	1 (1.8)	8 (7.9)	9 (5.8)
*eae*-ι1	2 (5.2)	8 (10.6)	0 (0.0)	0 (0.0)	2 (3.7)	8 (7.9)	10 (6.4)
*eae*-µR/ι 2§	0 (0.0)	0 (0.0)	0 (0.0)	0 (0.0)	0 (0.0)	0 (0.0)	0 (0)
*eae*- λ	**13 (34.2)**	**21 (28.0)**	**5 (33.3)**	**5 (19.2)**	**18 (33.9)**	**26 (25.7)**	**44 (28.5)**
*eae*-µB#	8 (21.1)	7 (9.3)	1 (6.6)	1 (3.8)	9 (16.9)	8 (7.9)	17 (11.0)
*eae*-νB#	2 (5.2)	1 (1.3)	0 (0.0)	0 (0.0)	2 (3.7)	1 (0.9)	3 (1.9)
*eae*-ξB#	0 (0.0)	2 (2.6)	0 (0.0)	0 (0.0)	0 (0.0)	1 (0.9)	2 (1.2)
*eae*-o	2 (5.2)	3 (4.0)	4 (26.6)	1 (3.8)	6 (11.7)	4 (3.9)	10 (6.4)

### Clinical characteristics of EPEC diarrhea by intimin types

Table 5 summarizes different clinical characteristics of severity according to intimin alleles in EPEC diarrheal cases. The most prevalent alleles (*eae*-λ and *eae*-β1), were mostly detected in children with fever and vomiting (three or more times per day), however, the proportions were not statistically significant. The duration of diarrhea varied from one to five days, with most of the cases resolving between 24 h and 3 days.

**Table 5:** Selected clinical characteristics and outcome (death) of EPEC diarrheal samples by intimin types

	Blood in stool			Fever			Vomiting (3 or more times per day)			Death		
Allele	Yes (5)	No (47)	*p* value	Yes (15)	No (37)	*p* value	Yes (27)	No (25)	*p* value	Yes (9)	No (43)	*p* value
	*n* (%)	*n* (%)		*n* (%)	*n* (%)		*n* (%)	*n* (%)		*n* (%)	*n* (%)	
*eae*-α1	0 (0)	1 (2.1)	0.904	0 (0)	1 (2.7)	0.712	0 (0)	1 (4)	0.481	1 (11.1)	0 (0)	0.370
*eae*-α2	1(20)	0 (0)	0.096	0 (0)	1 (2.7)	0.712	0 (0)	1 (4)	0.481	0 (0)	1 (2.3)	0.770
*eae*-β1	1 (20)	12 (25.5)	0.633	4 (26.6)	9 (24.3)	0.560	8 (29.6)	5 (20)	0.317	3 (33.3)	9 (20.9)	0.419
*eae*-ξR/β2B‡§	1 (20)	9 (19.1)	0.673	3 (20)	7 (18.9)	0.604	5 (18.5)	5 (20)	0.584	1 (11.1)	10 (23.2)	0.447
*eae*-δ/k/β2O||	0 (0)	1 (2.1)	0.904	0 (0)	1 (2.7)	0.712	1 (3.7)	0 (0)	0.519	0 (0)	1 (2.3)	0.823
*eae*-γ1	0 (0)	0 (0)	N/A	0 (0)	0 (0)	N/A	0 (0)	0 (0)	N/A	0 (0)	0 (0)	N/A
*eae*-θ/γ2	0 (0)	4 (8.5)	0.659	2 (13.3)	2 (5.4)	0.326	2 (7.4)	2 (8)	0.666	2 (22.2)	2 (4.6)	0.062
*eae*-ε1	0 (0)	2 (4.2)	0.815	1 (6.6)	1 (2.7)	0.498	0 (0)	2 (8)	0.226	0 (0)	2 (4.6)	0.674
*eae*-νR/ε2§	0 (0)	1 (2.1)	0.904	0 (0)	1 (2.7)	0.712	1 (3.7)	0 (0)	0.519	0 (0)	1 (2.3)	0.937
*eae*-ζ	0 (0)	1 (2.1)	0.904	0 (0)	1 (2.7)	0.712	1 (3.7)	0 (0)	0.519	0 (0)	1 (2.3)	0.674
*eae*-η1/η2	0 (0)	1 (2.1)	0.904	1 (6.6)	0 (0)	0.288	0 (0)	1 (4)	0.481	0 (0)	1 (2.3)	0.721
*eae*-η1	0 (0)	1 (2.1)	0.904	1 (6.6)	0 (0)	0.288	0 (0)	1 (4)	0.481	0 (0)	1 (2.3)	0.550
*eae*-ι1	0 (0)	2 (4.2)	0.815	0 (0)	2 (5.4)	0.512	1 (3.7)	1 (4)	0.735	0 (0)	2 (4.6)	0.513
*eae*-µR/ι 2§	0 (0)	0 (0)	N/A	0 (0)	0 (0)	N/A	0 (0)	0 (0)	N/A	0 (0)	0 (0)	N/A
*eae*- λ	1 (20)	17 (36.1)	0.428	8 (53.3)	10 (27)	0.070	10 (37)	8 (32)	0.465	3 (33.3)	15 (34.8)	0.499
*eae*-µB#	1 (20)	8 (17)	0.630	2 (13.3)	3 (8.1)	0.485	7 (25.9)	2 (8)	0.089	1 (11.1)	8 (18.6)	0.713
*eae*-νB#	0 (0)	2 (4.2)	0.815	0 (0)	2 (5.4)	0.502	1 (3.7)	1 (4)	0.735	1 (11.1)	1 (2.3)	0.177
*eae*-ξB#	0 (0)	0 (0)	N/A	0 (0)	0 (0)	N/A	0 (0)	0 (0)	N/A	0 (0)	0 (0)	N/A
*eae*-o	1 (20)	5 (10.6)	0.473	3 (20)	3 (8.1)	0.224	3 (11.1)	3 (12)	0.628	1 (11.1)	5 (11.6)	0.450

N/A – not applicable

## Discussion

In the present study, we assessed the allelic variability of the intimin virulence gene from a collection of EPEC isolates recovered from a case–control study of moderate-to-severe diarrhea (MSD) in children under five years in the Manhiça district, southern Mozambique, from 2007 to 2010. The findings demonstrate that, in this setting, the main burden of EPEC infections occurs in infants (aged less than 1 year), with typical EPEC predominating compared to atypical EPEC. However, the high prevalence of typical EPEC observed in the present analysis diverges with other studies conducted also in LMIC, where atypical EPEC was more prevalent compared to typical EPEC, including in some African settings [4, 18, 19, 25, 26]. These differences may be explained in part by the changes in the epidemiology of EPEC. Typical EPEC has been for many years considered to be more common in LMIC while atypical EPEC was associated with high income countries [16, 18]. Actually, lower prevalence of atypical EPEC was observed in a study conducted in Iranian children [3] and Nigeria [27]. The epidemiology change of atypical EPEC was report in East Delhi, India [18] however, the factors behind such variations remain unknown. Although the role of typical EPEC as an important cause of childhood diarrhea is well recognized, no significant association was detected between typical or atypical EPEC and MSD in the GEMS study [20]. Similar studies, found no clear association of EPEC with diarrhea, when comparing diarrheal samples and the control group [14, 28] including recent investigations in African populations [4]. Further genomic analyses, particularly whole-genome sequencing (WGS), could provide valuable insights into these discrepancies. WGS enables the identification of genomic features that may differentiate pathogenic from commensal or less virulent EPEC strains, such as the presence of additional virulence factors, regulatory elements, or mobile genetic elements influencing gene expression. WGS would also allow the identification of serotypes associated with these EPEC isolates. The traditional EPEC serogroups include O26, O55, O11, O114, O119, O125, O126, O127, O128, O142, and O158 [5, 29, 30], however, many other serotypes have been described in recent studies conducted in Brazil [31].

In spite of the high allelic variability observed, *eae*-λ, *eae*-β1, and *eae*-ξR/β2B‡§ were the most frequently detected intimin alleles, a finding in line with previous studies where *eae*-β1 and *eae*-λ have been reported as the most common alleles in human EPEC isolates [6, 14]. In addition, the typical EPEC isolates we describe had more diversity of intimin allele variants compared to the atypical ones, which may play a role in the differences in their virulence traits [24, 32, 33]. However, there were no clear statistical differences in the distribution of intimin alleles between diarrheal samples and the control group, which may be explained by the lack of association of the analyzed isolates with diarrhea [20]. This finding is further corroborated by the absence of significant differences in clinical characteristics of severity for the detected intimin alleles (Table 5). A similar study found that β- and kappa-intimin were the most prevalent among the episodes of longer duration of diarrhea and seem to be associated with clinical severity; however, the authors pointed out the need for further studies to evaluate additional virulence markers to determine which factors are crucial for disease [14]. These findings reinforce the idea that EPEC pathogenicity could be a combination of several virulence factors [34]. Some important virulence encoding genes, include the heat-stable toxin *astA* coding for EAST1 [25, 35, 36], adhesins and effector proteins such as *fimA*, *lifA*, *espA*, *espB*, *espC*, *espD* [37]. Furthermore, it has been suggested that interpreting the relationships between intimin or other virulence genes and clinical characteristics could be complicated by many factors, such as protectors in breast milk, previous infection with related intimin types, infection onset, genes associated or not associated with intimin in a given strain, and genetic predisposition of the host [14]. Thus, comparative genomic approaches could reveal lineage-specific variations, novel intimin alleles, or horizontally acquired genes that contribute to the observed differences in prevalence and pathogenic potential between isolates recovered from cases and controls.

The data we present should be interpreted considering some limitations. Although the PCR used to determine the allelic variability [24], proved to be efficient and reproducible, allowing the typing of a large number of isolates (131 out of 154) in a simple and rapid manner, some alleles could not be typed. Thus, further studies are needed to evaluate additional virulence markers to determine which factors are crucial for disease. Therefore, future analysis and especially using whole-genome sequencing to assess the virulence factors and lineages will be necessary to gain more insights into the role of EPEC in diarrheal disease, especially in the African settings. To our best knowledge, this is the first study characterizing the intimin virulence gene in Mozambique.

## Conclusions

This study confirmed a high diversity of intimin alleles among EPEC diarrheal isolates from a rural area in southern Mozambique. Although there were no clear associations of intimin alleles with clinical characteristics of severity, some variants were more common in certain characteristics, suggesting a possible role for disease severity. Further studies are needed to evaluate additional virulence markers to determine which factors are crucial for disease, associated serogroups and genetic lineages.

## Data Availability

All the data generated or analyzed through this study are included in this published article.

## Abbreviations

EPEC: Enteropathogenic Escherichia coli
LMIC: Low-Middle-Income Country
AE: Attaching and Effacing
LEE: Locus for Enterocyte Effacement
STEC: Shiga-Toxin producing E.coli
LA: Localized Adherence
EAF: EPEC Adherence Factor
Bfp: Bundle forming pilus
GEMS: Global Enteric Multicenter Study
MSD: Moderate-Severe Diarrhea
CISM: Centro de Investigação em Saúde de Manhiça
